# An algorithm applied to national surveillance data for the early detection of major dengue outbreaks in Cambodia

**DOI:** 10.1371/journal.pone.0212003

**Published:** 2019-02-07

**Authors:** Julia Ledien, Kimsan Souv, Rithea Leang, Rekol Huy, Anthony Cousien, Muslim Peas, Yves Froehlich, Raphaël Duboz, Sivuth Ong, Veasna Duong, Philippe Buchy, Philippe Dussart, Arnaud Tarantola

**Affiliations:** 1 Epidemiology and Public Health Unit, Institut Pasteur du Cambodge, Phnom Penh, Cambodia; 2 Centre National de Malariologie (CNM), Phnom Penh, Cambodia; 3 Mathematical Modelling of Infectious Diseases Laboratory, Institut Pasteur, Paris, France; 4 UMR ASTRE CIRAD INRA, Montpellier, France; 5 Virology Unit, Institut Pasteur du Cambodge, Phnom Penh, Cambodia; 6 GlaxoSmithKline, Vaccines R&D, Singapore, Singapore; 7 Epidemiology unit, Institut Pasteur de Nouvelle-Calédonie, Nouméa, New Caledonia; The University of Hong Kong, CHINA

## Abstract

Dengue is a national priority disease in Cambodia. The Cambodian National Dengue Surveillance System is based on passive surveillance of dengue-like inpatients reported by public hospitals and on a sentinel, pediatric hospital-based active surveillance system. This system works well to assess trends but the sensitivity of the early warning and time-lag to usefully inform hospitals can be improved. During The ECOnomic development, ECOsystem MOdifications, and emerging infectious diseases Risk Evaluation (ECOMORE) project’s knowledge translation platforms, Cambodian hospital staff requested an early warning tool to prepare for major outbreaks. Our objective was therefore to find adapted tools to improve the early warning system and preparedness. Dengue data was provided by the National Dengue Control Program (NDCP) and are routinely obtained through passive surveillance. The data were analyzed at the provincial level for eight Cambodian provinces during 2008–2015. The R surveillance package was used for the analysis. We evaluated the effectiveness of Bayesian algorithms to detect outbreaks using count data series, comparing the current count to an expected distribution obtained from observations of past years. The analyses bore on 78,759 patients with dengue-like syndromes. The algorithm maximizing sensitivity and specificity for the detection of major dengue outbreaks was selected in each province. The overall sensitivity and specificity were 73% and 97%, respectively, for the detection of significant outbreaks during 2008–2015. Depending on the province, sensitivity and specificity ranged from 50% to 100% and 75% to 100%, respectively. The final algorithm meets clinicians’ and decisionmakers’ needs, is cost-free and is easy to implement at the provincial level.

## Introduction

Dengue fever is an infectious disease transmitted by the bite of mosquitoes (*Aedes* spp) and caused by a virus of the genus *Flavivirus*. Dengue viruses are divided into four antigenically distinct serotypes (DENV-1, DENV-2, DENV-3 and DENV-4). Approximately 96 million symptomatic dengue infections occurred worldwide in 2010 [1]. Most infections are benign but severe forms requiring hospital admission include dengue hemorrhagic fever (DHF) and dengue shock syndrome (DSS), occurring in approximately 1/1,000 infections [2]. The case-fatality rate (CFR) for severe dengue ranges from 20% in some outbreaks to less than 1% [2].

The burden of dengue is also socio-economic. The overall cost of dengue fever was estimated at $39 billion USD per year, including medical care, surveillance, vector control, and lost productivity [2]. A 2016 estimate reported that dengue was responsible for the loss of 1.14 million (0.73–1.98 million) Disability-Adjusted life years (DALYs) in 2013, representing a 58% increase from 1990 [3].

The Asia-Pacific region where the four dengue serotypes circulate is particularly affected with nearly 75% of its population exposed to dengue [4]. In 2012, WHO estimated that 356,838 dengue cases were reported in the Western Pacific Region, with a case-fatality rate (CFR) of 0.34% [5]. Another study found a global age-standardized incidence of 34.3 (12.7–75.0) cases per 1,000 people with an age-standardized mortality rate of 8.49 per million person-years in 2013 [3]. In twelve Southeast Asian countries, the aggregate annual economic burden of dengue was estimated at US$950 million in which productivity loss represents 52% of the total amount [4].

Cambodia has one of the highest documented dengue burdens per capita in Southeast Asia. Dengue is endemic and seasonal dengue peaks occur each year during the rainy season, usually between June and October [6]. On average, the sentinel surveillance system reported 103 cases per 10,000 population with a CFR of 1% to 2% annually since 2000 [7]. Since 2002, 30% to 60% of dengue-like syndromes reported are DHF and DSS [7] for which hospitalization, careful patient management and intravenous rehydration are needed.

Depending on the immunological status of the population and the DENV serotypes/strains emergence and circulation, major outbreaks can occur in Cambodia [6]. In 2007, during a DENV-3 outbreak, a fever surveillance study compared dengue incidence in rural and urban areas and found respectively incidences of 71/1,000 person-seasons and 17/1,000 person-seasons, respectively [4].

Unlike seasonal peaks, major outbreaks are not easily predicted, leading to hospital overcrowding and a decrease in quality of care, especially for severe cases because of medication shortages and hospital staff exhaustion, particularly in provincial hospitals where resources are particularly scarce [4]. Without early warning system, when a major outbreak occurs, the number of patient needed care is much higher than the expectation and there are not enough beds, not enough medicine in stock and the staff is rapidly enabled to provide care to everyone. Timely prediction of major dengue outbreaks is, therefore, an important issue in Cambodia, as improved but targeted health system preparedness could improve hospital care despite a massive increase in dengue patient admissions.

The National Dengue Surveillance System includes: 1/ A passive surveillance of dengue-like pediatric inpatients reported by public hospitals to the Communicable Diseases Center of the Ministry of Health (CDC-MoH); and 2/ a sentinel, pediatric hospital-based active surveillance system managed by the National Dengue Control Program (NDCP) of the National Center for Malariology (CNM), Ministry of Health. A subset of these pediatric patients is tested each week at Institut Pasteur du Cambodge (IPC) to ensure laboratory confirmation and virological surveillance [7].

With IPC support, CNM provides a weekly report to dengue stakeholders documenting weekly and monthly national incidences by the administrative division as well as ongoing outbreaks [7]. The existing system uses weekly data. A major outbreak is defined as a significant rise in the incidence beyond the estimated endemic level. Although this method is widely used and recommended by WHO, it raises several issues related to outbreak definitions. Indeed, this is currently set at two standard deviations from endemic levels although the latter is very difficult to quantify [8]. In Cambodia, the incidence for the five previous years (excluding years with major outbreaks) is considered the endemic backdrop. If the national incidence crosses this threshold, the National Dengue Program is tasked to undertake vector control interventions in the outbreak areas [7]. This system lacks sensitivity for variability at the hospital level, a scale at which resources are used to manage patients [8].

More comprehensive analytic methods have been developed which have proved their effectiveness and timeliness [9]. More specifically, outbreak detector algorithms have been shown to be simple, efficient and easy to use [10].

The ECOnomic development, ECOsystem MOdifications, and emerging infectious diseases Risk Evaluation (ECOMORE) project is a collaborative project implemented in four countries (Myanmar, Vietnam, Cambodia and Lao PDR). Its overall objectives are to better understand anthropogenic ecological changes responsible for the emergence of infectious diseases and to measure the health risks for local communities as a result of improvement of surveillance systems and strengthening of national and regional cooperation. More specifically, the Cambodian component aimed to stimulate and federate a dynamic of national collaborations to strengthen the Cambodian surveillance and early warning capacity of emerging-vector-borne disease, using Chikungunya and Dengue as proxies.

As part of the ECOMORE project, this study was designed to provide a major dengue outbreak detection tool, providing a more flexible system to forecast outbreaks rather than describe the ongoing situation, providing early warning to guide preparedness efforts at the local level. This kind of tool is essential to better manage major outbreaks at the hospital level through staff, medication and bed allocation. Indeed, at the hospital level, a significant amount of patients flocking at the same time provokes disruption in hospital functioning and a decreased quality of care. Regarding dengue, patient clinical monitoring is the most important element to prevent severe cases and decrease CFR. Hospitals must have enough beds and enough staff to care for dengue patient and this is only possible if hospital managers are aware of the possible occurrence of a major outbreak.

## Material and methods

Firstly, as the surveillance system only detects suspected dengue cases, it is important to ensure that the pattern observed on dengue-like syndrome reflects real dengue cases. All probable and laboratory-confirmed dengue cases are reported for the previous week, according to the case definition provided by CNM’s National Dengue Control Program (NDCP) [7]. At IPC, virological and serological analyses are performed for the first suspected dengue case five days a week (five samples tested in each of eight sentinel hospitals every week) to detect new circulating serotypes and evaluate the proportion of confirmed dengue among suspected cases, using methods described elsewhere [6]. Surveillance data, as well as laboratory-confirmed cases in the eight provinces studied, were aggregated by weeks from 2012 to 2015 in order to increase the number of cases represented over 51 weeks. We verified that dengue syndromic data 2012–2015 was well correlated with laboratory surveillance data using Pearson’s correlation coefficient.

The anonymized dataset is constituted by the weekly number of suspected dengue cases from 2004 to week 40 of 2015 residing in provinces documented by the ECOMORE-supported CNM sentinel pediatric hospital wards in eight of Cambodia’s 23 provinces: Phnom Penh,Kampong Speu, Kampong Chhnang, Kratie, Kampot, Takeo, Kampong Cham and Battambang Provinces. Pediatric inpatients with suspected dengue are systematically documented for age, district and province of residence as well as the date of symptoms onset.

Several early warning methods exist but have not be found to be effective to predict major dengue outbreak at a local scale. Detection of abnormalities in time series is a method that can handle the seasonal variations and flags abnormal amount of cases. Thus, major outbreaks rather than seasonal outbreaks are detected. These methods apply algorithms on surveillance time series and can be updated by added new weekly or daily case-count.

The Surveillance R-package [11] provides automated algorithms for the detection of abnormalities in time series of the class of the reference value based. The surveillance algorithm is a procedure repeated at each time point where the reference values are used to calculate a theoretical value which is then compared with the observed value. The process works like a sliding window where the reference values are observed values of the previous weeks and values at the time point studied of the previous years. The time point studied is flagged when the observed value is significantly higher than the theoretical one.

We compared four outbreaks detection algorithms applied to weekly dengue surveillance data to detect abnormalities for the current week in the time series (the Centers for Diseases Control and Prevention (CDC) method [12]; the Farrington method [13]; The Robert Koch Institute (RKI) method [14]; And a Bayesian approach [15]). The Bayesian approach provided better results and is, therefore, the only one presented in this paper. We applied the algorithm at the provincial level, as all provinces may be not affected by dengue at the same time and with the same intensity. The algorithms are all based on the construction of a predictive distribution for the number of reported cases for the current week of the year ***t*** in each province [16]. The latter is obtained using the reported cases from the ***b*** previous years: The ***w***_***0***_ previous weeks before ***t*** in the current year and the ***w*** weeks around ***t*** in the previous years. This selection takes into account the seasonality in the epidemic by considering only relevant data (i.e. data obtained in similar seasons). Once this distribution is obtained, an upper limit for the cases count of the current week—i.e. the number of cases above which the observed count is considered abnormal—is set as being greater than the 95^th^ percentile of this distribution. An outbreak threshold was defined for each Province based on the annual dengue peak for the eight preceding years, to detect case counts higher than the normal seasonal peak. Depending on the value attributed to the threshold, seasonal, major or even exceptional outbreak could be detected. A variable was added to each week of the database to indicate whether the threshold had been crossed. This information is used to assess whether the algorithms effectively flagged an abnormality before the occurrence of an outbreak, providing sensitivity and specificity of the algorithm. The sensitivity is the probability of a flag given that an outbreak occurs and the specificity is the probability of no alarm, given that an outbreak does not occur [10]. Also, the average time elapsed between the flag and the outbreak is calculated.

In order to find the most suitable tool, each provincial algorithm was assessed using the following parameters:

- ***b*** (number of previous years to include) = 1, 2, 3, 4, 5, 6, 7, and 8
- ***w*** (for the previous year, the number of weeks to include around the week we are predicting) = 1, 2, 3 and 4
- ***w***_***0***_ (for the current year the number of previous weeks to include) = 1, 2, 3 and 4

For each Province, the optimal set of parameters was selected using the Euclidean distance between the sensitivity and 1*-*specificity. The best algorithm selected in each province was then used to build a yearly major outbreak sensor, documenting successive flagged aberrations and not only isolated caseload aberrations. Our ECOMORE-CNM dengue outbreak sensor was tested using the following criteria: We considered an alarm only if multiple rises in cases occurred (more than two “aberrations” in four weeks) before the dengue epidemic. Performance of these sensors was then assessed by calculating sensitivity and specificity for the detection of major outbreaks on the period 2008–2015 (no information is available for 2004–2007 period because the algorithms need the reference values of the previous years). The average time between alarm and outbreak occurrence in the hospital was also computed. More information about the method is provided in S1 File.

Weekly sentinel hospital surveillance data was entered at CNM and graphs were generated using Excel (Microsoft Excel, Redmond, Washington, USA). Analyses were performed using the R statistical software (R Foundation for Statistical Computing, Vienna, Austria.) [17] and the “Surveillance” R-package [11]. The ECOMORE project received approval from the National Ethic Committee for Health Research of Cambodia (approval #0144 NECHR dated 26 August, 2013) and was funded by the Agence Française de Développement.

## Results

**Fig 1:**
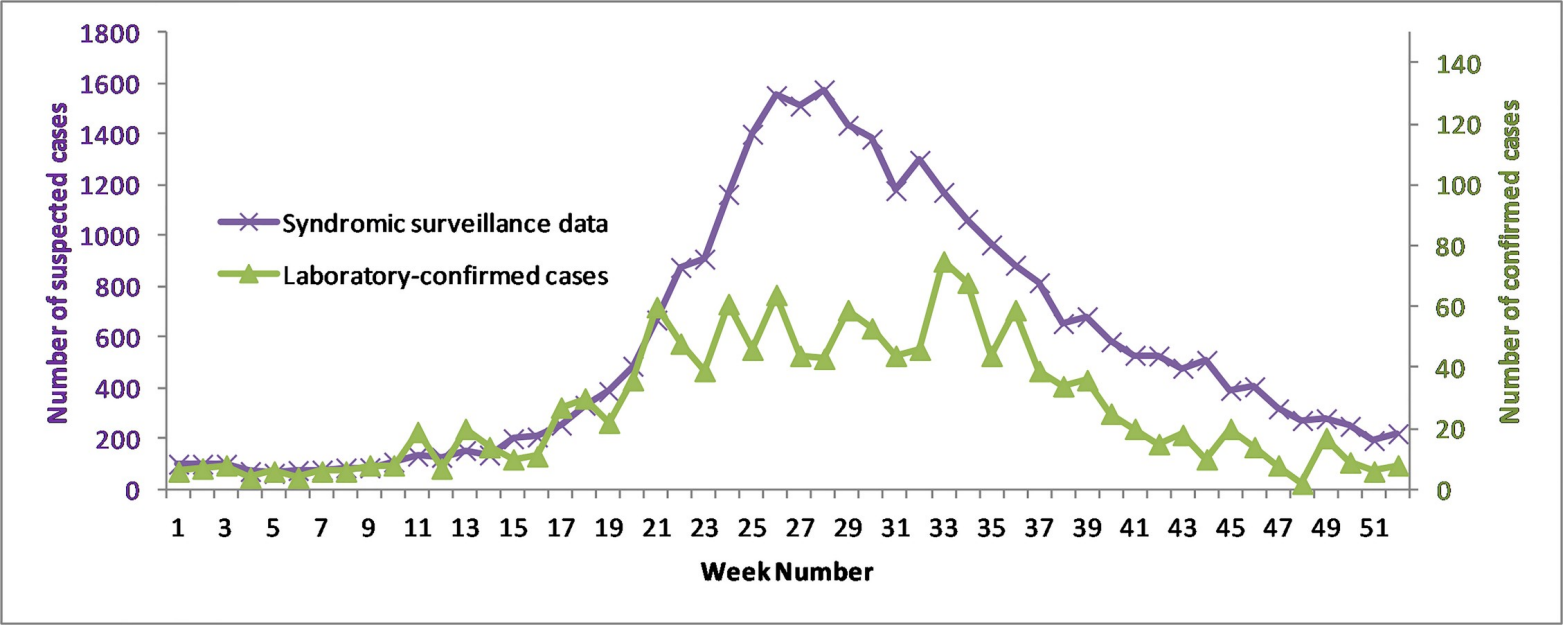
Dengue-like syndromes reported to CNM and confirmed by laboratory methods at IPC between 2012–2015 from the sentinel sites(Battambang, Kampot, Kampong Speu, Kampong Chhnang, Kampong Cham, Kratie, Phnom Penh and Takeo referral hospitals) aggregated by week.

Fig 1 presents the aggregated results of virological surveillance performed by IPC during 2012–2015.

The trend of laboratory-confirmed cases closely matches that of the dengue-like syndrome surveillance for 2012–2015 in the eight ECOMORE Provinces (Pearson's product-moment correlation coefficient r = 0.87 [0.78; 0.92], p<2.2x10^-16^).

Table 1 presents the parameters, sensitivity and specificity of the best algorithm of each province. The algorithms showed good sensitivity and specificity in all eight provinces, ranging from 75% to 100% sensitivity and from 89% to 96.5%, specificity, depending on the province.

**Table 1:** Parameters, sensitivity and specificity of the optimal Bayesian dengue sensor algorithm for the eight ECOMORE sentinel sites, 2012–2015, Cambodia.

Province	Outbreak weekly threshold (n)	b*	w**	w_0_°	Se • (%)	Sp¤ (%)	Dist^◊^
Kampong Cham	120	3	3	2	86.21	89.04	0.18
Phnom Penh	80	3	3	3	84.38	93.09	0.17
Kratie	10	3	3	3	76.19	96.51	0.24
Takeo	60	3	3	3	100	90.42	0.10
Kampong Speu	60	3	3	3	95.65	90.84	0.10
Kampong Chhnang	40	3	3	3	75.00	93.17	0.26
Battambang	20	3	3	2	94.55	93.81	0.08
Kampot	20	3	3	4	86.21	93.15	0.15

* ***b =*** number of previous years to include

** ***w*** = for the previous year, the number of weeks to include around the week we are predicting

° ***w***_***0***_ = for current year the number of previous weeks to include

• Se = Sensitivity True Positives/(False Negatives + True Positives)

¤ Sp = Specificity True Negatives/ (True Negatives + False Positives)

^◊^ Dist = Euclidean distance between Se and 1-Sp.

**Fig 2:**
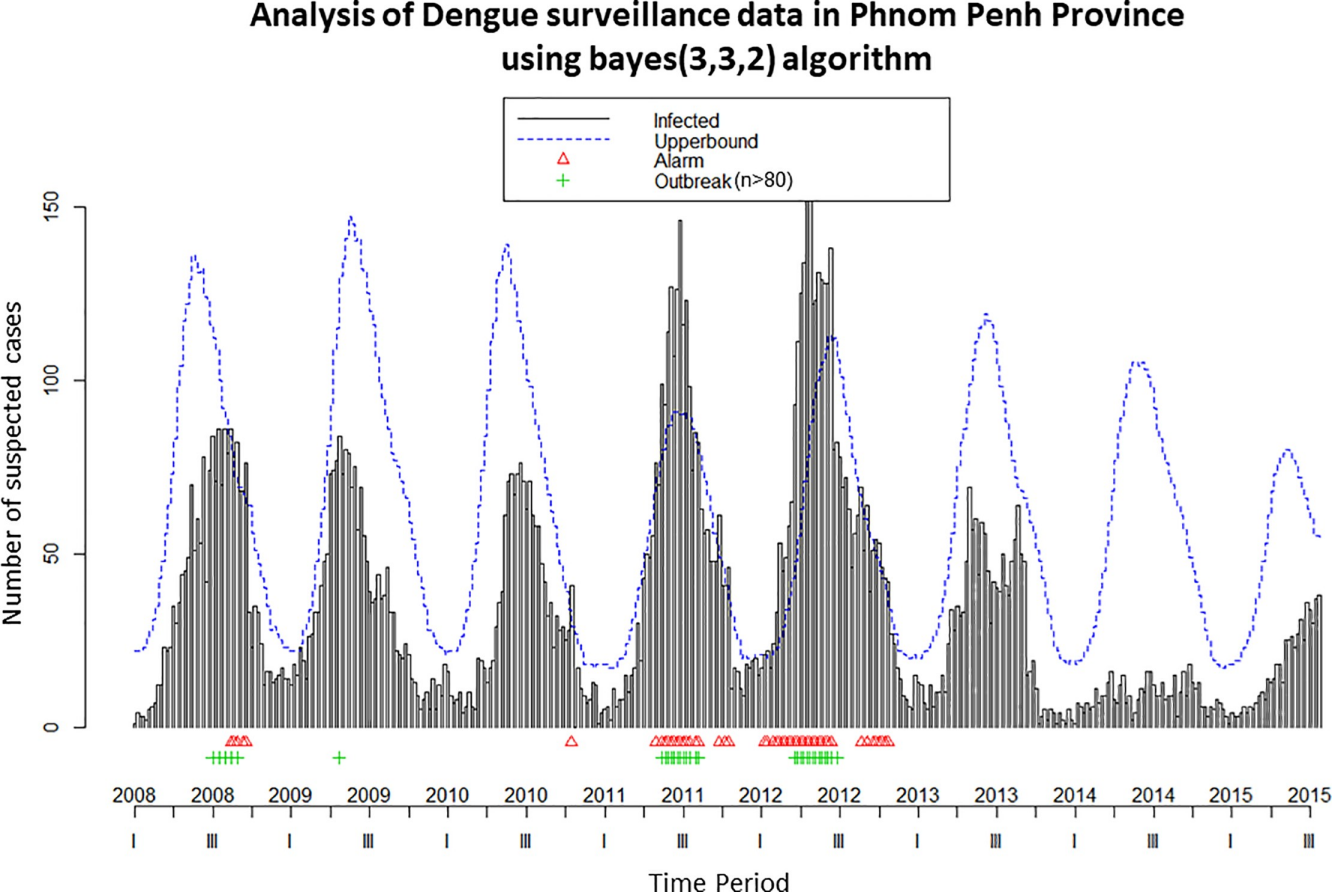
Dengue surveillance graphic of Phnom Penh province using the surveillance R-package Bayesian method with their parameters (b, w, w_0_), Cambodia, 2004–2015.

Note: Data from 2004 are used by the algorithm but only those from 2008 are displayed. The number of cases observed in Phnom Penh from 2008 to 2015 is shown as grey bars. The green “plus” sign corresponds to the outbreak threshold defined. The blue dotted line is the upper bound of the 99% confidence intervals calculated over the previous years with the predictive values by the algorithm. If the observed values cross the blue line, a red triangle appears, signalling an aberration in the seasonality. **b =** number of previous years to include; **w** = for the previous year, the number of weeks to include around the week we are predicting; **w**_**0**_ = for current year the number of previous weeks to include.

Fig 2 presents an example of the graph of surveillance data and the detection of anomalies obtained with Surveillance R-Package when running the algorithm selected for Phnom Penh province.

In 2008 the red triangles appeared after the outbreak (marked by green plus signs). This means the major outbreak alarm would have been raised too late. In 2009, the algorithm missed the major outbreak. In 2011 and 2012, the red triangles appeared a few weeks before the major outbreaks. In 2011 and 2015 some red triangles appeared and a seasonal epidemic but there was no major outbreak (a “false-positive”) therefore the algorithm did not perform well. In 2010, 2013 and 2014, the algorithm correctly detected that there was no major outbreak. For these 8 years, the algorithm therefore predicted wrongly or too late in four instances. The graphics for the other provinces studied are presented in S1 Fig and S2 Fig.

The flags raised by the algorithm cannot be used on their own to predict major outbreaks. Epidemiological interpretations are necessary to take into account the context. The rules applied are the following: We considered an alarm only if multiple raises in cases occurred (more than two “aberrations” in four weeks) before the dengue epidemic. Based on the flags raised by the algorithm and epidemiological interpretations, the ECOMORE-CNM dengue outbreak sensor was applied to each province. For each year studied and each province, the prediction provided was compared to observed data. Table 2 summarizes its performance over eight years in each province. The resulting Dengue Outbreak Sensor has an overall sensitivity of 73% and a specificity of 97% in predicting large epidemics. The average time lag between the detection of an important dengue fever outbreak and its occurrence was 5.2 ± 5.0 weeks.

**Table 2:** Characteristics of the ECOMORE-CNM dengue outbreak sensor by province studied for eight years (2008–2015), Cambodia.

Province	TP* (n)	FP** (n)	TN° (n)	FN^●^ (n)	PPV’ (%)	NPV” (%)	Se^□^ (%)	Sp^◊^ (%)	AvT^Δ^(n)
Battambang	3	0	4	1	100	80	75	100	3
Kampot	2	0	5	1	100	83	67	100	9
Kampong Chhnang	4	0	3	1	100	75	80	100	6.5
Kampong Cham	3	1	3	1	75	75	75	75	5
Kampong Speu	2	0	5	1	100	83	67	100	7.5
Katie	2	0	5	1	100	83	67	100	3.5
Phnom Penh	2	0	4	2	100	67	50	100	4.5
Takeo	2	0	6	0	100	100	100	100	2.5
Overall	22	1	35	6	96	85	72.6	96.9	5.2 (sd = 5.0)

* TP: True Positive, number of years during which the alarm was flagged and the outbreak occurred

** FP: False Positive, number of years during which the alarm was flagged but the outbreak did not occur

^○^ TN: True Negative: number of years during which the alarm was not flagged and the outbreak did not occur

FN^●^: False Negative, number of years were the alarm was not flagged but the outbreak occurred

PPV’: Positive Predictive Value, TP/(TP+FP)

NPV”: Negative Predictive Value, TN/(TN+FN)

Se^□^: Sensitivity, TP/(TP+FN)

Sp^◊^: Specificity, TN/(TN+FP)

AvT^Δ^: average time in week elapsed between alert and outbreak

## Discussion

The ECOMORE project aims to create a national dynamic around dengue surveillance, helping to further improve public health by developing an early warning system. This was a request from hospital staff, wishing to be informed a few weeks in advance of an important outbreak so they may prepare accordingly.

Surveillance has been defined as “the ongoing systematic collection, collation, analysis and interpretation of data; and dissemination of information to those who need to know in order that action may be taken” [18]. In short: appropriate information for timely and adequate action. Although a dengue vaccine is now licensed in some countries, it is not yet accessible to Cambodians and there is, to date, no specific treatment for dengue fever. Furthermore, the dengue vaccine available to date is not recommended in children aged below nine [19], by which age most have already undergone two DENV infections in Cambodia. Rehydration fluids and consumables can be mobilized to prevent or deal with Dengue Shock Syndrome to ensure appropriate clinical management and reduce the case fatality rate of severe dengue to less than 1% [4]. The dengue CFR is lowered through monitoring and adequate fluid management [20]. Surveillance and early detection of outbreaks may, therefore, prevent deaths by guiding local preparedness, especially in healthcare structures. Hospitals order and prepare a number of consumables each year. In Cambodia, these are requested on the basis of a “normal” outbreak and need to be rapidly complemented in case of a sudden increase in demand. Early outbreak detection may also prevent hospital overload. If an important outbreak is identified with enough foresight further actions can be taken at several levels: the hospital management team can prepare staff through information and training; reorganize work schedules and/or transfer of additional staff to the pediatric unit; Additional beds and mosquito nets can be prepared for incoming patients; Hospital grounds can be cleaned up to avoid mosquito breeding inside or around the hospital area in order to prevent dengue transmission in the hospital.

An early warning system should be sensitive enough to predict an outbreak and specific enough to avoid false alarms liable to cause wasteful redirection of human and other resources. It also should be able to analyze non-specific signals, especially “syndromic” data [10].

In Taiwan, an approximate entropy algorithm combined with pattern recognition was used to predict dengue fever outbreaks several weeks prior to their occurrence. The authors used two patterns and obtained a sensitivity of 68% and a specificity of 54% with the first and, a sensitivity of 90% and a specificity of 46% with the second. Their method was able to predict the occurrence of an outbreak 3.1 ± 2.2 weeks in advance and 2.9 ± 2.4 weeks in advance for the first and second pattern, respectively [21]. A Malaysian team developed an algorithm based on danger theory using climatic data in addition to referrals and dengue registered cases, with an average sensitivity of 77% and an average specificity of 99% [22].

We used the Surveillance R-package to see whether we could predict major outbreaks to support healthcare providers. The ECOMORE-CNM yearly Dengue Major Outbreak Sensor, based on the Surveillance R-package is a highly performant tool to predict major incoming dengue outbreaks in Cambodia. Its high specificity provides important information at the local level: If no alarm sounds off before or at the beginning of the Dengue season, there is 96.9% probability that there will not be any major outbreak that year. Although it varies from one province to another, its sensitivity is also high (72.6%). This means that hospitals can be informed of a major outbreak with a high degree of confidence more than one month in advance, on average 5.2 ± 5 weeks. Some alarms rang without justification but these were mostly isolated and/or at the end of the dengue season. The alarms that should be taken into consideration are the ones occurring repeatedly (two or more anomalies in a few weeks) before the dengue season begins. Alarms accurately predicting a major outbreak rang every week since the beginning of that outbreak.

We chose to not merge all locations because these might have overlapped and masked each other. Indeed, outbreaks did not occur throughout Cambodia at the same time and with the same intensity [23]. The automatized early-warning tool we developed should, therefore, better be conducted at each provincial level rather than the national level. An algorithm can be used, taking into account the spatial distribution of the sentinel sites, but these are time-consuming and require specially-trained staff and frequent updating [10].

Another important issue is that data quality influence algorithms’ performance. If the cases are not clinically well documented or if the case definition is not respected, the number of cases is under- or overestimated. For this reason, the joint CNM-IPC ECOMORE project ensured the quality of the data collected in the sentinel sites through evaluations, training and knowledge translation. The quality of the data provided by non-sentinel sites, however, was not evaluated. Should the outbreak sensor be extended to all of Cambodia, syndromic surveillance data quality will need to be assessed (12). Our laboratory surveillance data, however, shows that clinicians know dengue well and that syndromic surveillance is a good proxy for dengue circulation in Cambodia.

Dengue is essentially a childhood disease in Cambodia [24]. This could have led to a selection bias because clinicians are more likely to suspect dengue if the patient is young considering the high incidence among children. Dengue is rarely suspected in febrile disease in Cambodian adults, in which case the patient is not reported in the dengue system. In Cambodia, however, the four DENV serotypes circulate with different intensity depending on the year and a significant proportion of the Cambodian population may well have undergone infections with the four serotypes before reaching adulthood. The issue of dengue surveillance in Cambodia being based principally in pediatric sites is therefore likely marginal.

The main limit of our study is that the algorithm used needs to be trained, which may cause a loss of robustness in early phases of surveillance, or if the outbreak pattern changes or differs significantly from previous years. The 2015 outbreak in Kampong Chhnang province, for instance, was reported later than usual and was not detected in a timely way by our algorithm [22]. Robustness could also be lower because the method requires a sample of outbreak and non-outbreak periods. If any change occurs in the sensor’s outbreak definition, the algorithm would need to be reviewed or changed entirely [22]: Because of the learning phase, the algorithm cannot face rapid changes in epidemic patterns. Such changes, however, are unlikely in the short/medium term in Cambodia.

## Conclusion

Surveillance algorithms can be implemented easily and free of charge using the Surveillance R-package. This robust and effective tool can easily be trained and used by adding new data and processing commands on a weekly basis. As part of the ECOMORE project, it is currently being transferred by IPC to CNM staff in charge of compiling weekly reports and sending them to the referral hospital staff and decisionmakers before and during the dengue season. This tool is simple, free of charge and its use will help manage dengue outbreak by informing hospital staff, potentially preventing overload and anticipating drug and consumable needs. Time-space trends monitoring may also help highlight changing patterns of the epidemic or help identify new risk factors [9]. Further work is ongoing on dengue modeling by the ECOMORE team to better understand the dynamics of dengue in Cambodia and its spread along roads to potentially develop innovative countermeasures.

## Supporting information

S1 File Model used for outbreak detection. (DOCX)

S1 Fig **Dengue surveillance graphics using the Bayesian method with their parameters (b, w, w**_**0**_**), in a) Battambang, b) Kampot, c) Kampong Chhnang and d) Kampong Cham provinces, Cambodia, 2004–2015. b =** number of previous years to include; **w** = for the previous year, the number of weeks to include around the week we are predicting; **w**_**0**_ = for current year the number of previous week to include. A red triangle appears when the number of cases crosses the upper limit of the predictive curve.
(TIF)

S2 Fig **Dengue surveillance graphics using the Bayesian method with their parameters (b, w, w**_**0**_**), in a) Kampong Speu, b) Kratie and c) Takeo provinces, Cambodia, 2004–2015. b =** number of previous years to include; **w** = for the previous year, the number of weeks to include around the week we are predicting; **w**_**0**_ = for current year the number of previous week to include. A red triangle appears when the number of cases crosses the upper limit of the predictive curve.
(TIF)
