# Supplementary material for: An algorithm applied to national surveillance data for the early detection of major dengue outbreaks in Cambodia
Source: PLoS One. 2019 Feb 7;14(2):e0212003. doi: 10.1371/journal.pone.0212003 (PMC6366704; doi:10.1371/journal.pone.0212003)
Supplement: S1 File — (DOCX) [file pone.0212003.s001.docx]

**Supporting Information File 1: model used for outbreak detection**

To detect abnormalities in outbreaks, we compared four algorithms implemented in the R “Surveillance” package. All the algorithms are based on the same idea, which is the use of the number of cases observed during the previous years for a given week to construct an expected (“predictive”) distribution of the number of cases observed for the current year and the corresponding week. The algorithms can be divided into 3 steps:

*Step 1*: Construction of a predictive distribution from a set of reference values $R(w, w0, b)$for the number of reported cases for the current week of the year $t$, using the reported cases from the $b$ previous years

- - the $w0$ previous weeks before $t$ in the current year
  - the $w$ weeks around $t$in the previous years

Let $y_{0,t}$ be the number of reported cases during week$t$of the current year and $y_{-i,t}$ be the number of reported cases during week$t$ for the $i$^th^  year in the past (1). We have:

$$R\left( w, w0, b \right)= \left( \bigcup_{i=1}^{b} \bigcup_{j=-w}^{w} y_{-i,t+j} \right)\cup\left( \bigcup_{k=-w0}^{-1} y_{0,t+k} \right)$$

*Step 2:* The number of cases observed is considered abnormal (aberrations) for the current year and current week ≥ 95th percentile of this distribution.

*Step 3:* We considered an alarm only if multiple aberrations occurred (more than two aberrations in 4 weeks).

- We compared several methods to construct the predictive distributions from the reference values $R\left( w, w0, b \right)$. First, the Centers for Diseases Control and Prevention (CDC) method, which use a simple Gaussian model. Second, the Farrington method, which uses a quasi-Poisson distribution. Third, the Robert Koch Institute (RKI) method, which assumes a Gaussian or a Poisson based on the mean of the cases counts for the previous years. Finally, we also used a Bayesian approach, assuming a negative-binomial distribution with parameters estimated through a Bayesian procedure (2). We compared the models with the following parameters :
  - - $b$ = 1, 2, 3, 4, 5, 6, 7, and 8
    - $w$ = 1, 2, 3 and 4
    - $w0$ = 1, 2, 3 and 4

A detailed description of each method can be found in the references given above.

- For each Province, the optimal algorithm and set of parameters was selected using the Euclidean distance between the sensitivity and 1-specificity, with a real major outbreak defined according to expert-based thresholds.

References:

1. Hohle M, Riebler A, Paul M. The R-Package ’surveillance’ [Internet]. CRAN R-project; 2015 [cited 2015 Oct 23]. Available from: https://cran.r-project.org/web/packages/surveillance/vignettes/surveillance.pdf

2. Monitoring Count Time Series in R: Aberration Detection in Public Health Surveillance | Salmon | Journal of Statistical Software. [cited 2018 Sep 26]; Available from: https://www.jstatsoft.org/article/view/v070i10
