# Supplementary figures and images for: An algorithm applied to national surveillance data for the early detection of major dengue outbreaks in Cambodia
Source: PLoS One. 2019 Feb 7;14(2):e0212003. doi: 10.1371/journal.pone.0212003 (PMC6366704; doi:10.1371/journal.pone.0212003)

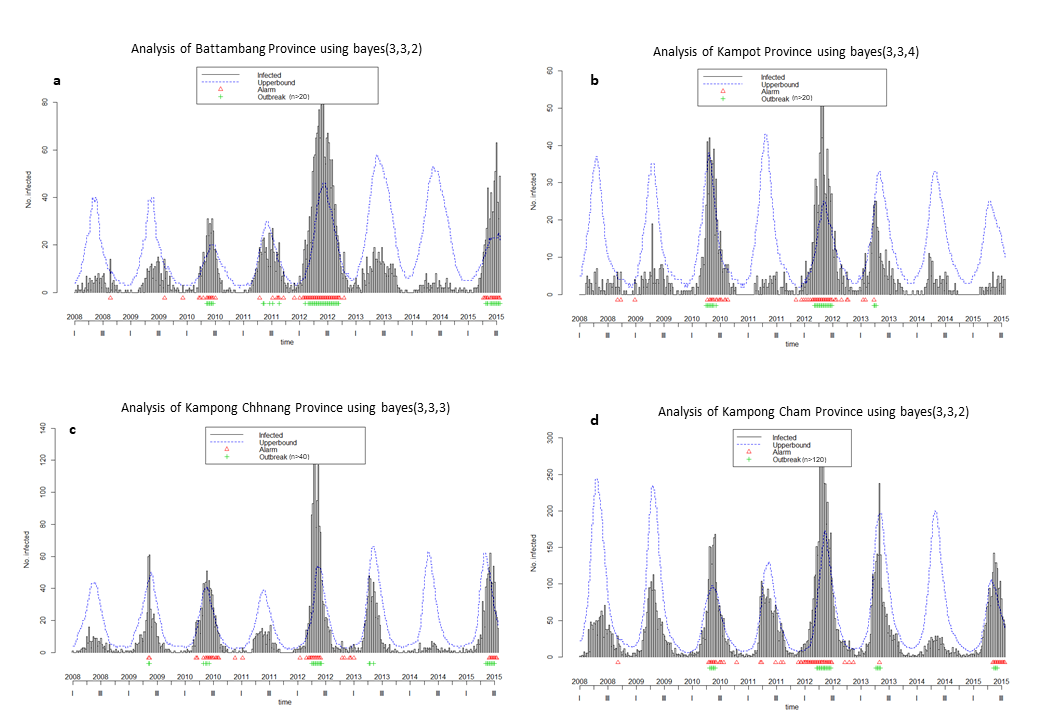

Supplement: S1 Fig — Dengue surveillance graphics using the Bayesian method with their parameters (b, w, w0), in a) Battambang, b) Kampot, c) Kampong Chhnang and d) Kampong Cham provinces, Cambodia, 2004–2015. b = number of previous years to include; w = for the previous year, the number of weeks to include around the week we are predicting; w0 = for current year the number of previous week to include. A red triangle appears when the number of cases crosses the upper limit of the predictive curve. (TIF) [file pone.0212003.s002.TIF]

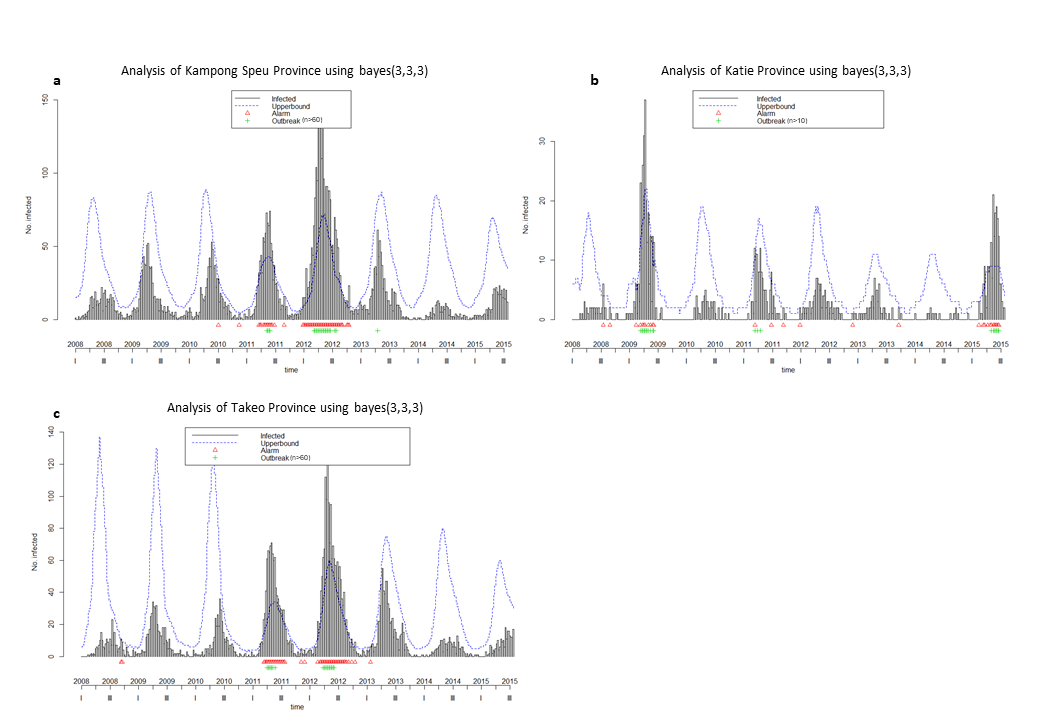

Supplement: S2 Fig — Dengue surveillance graphics using the Bayesian method with their parameters (b, w, w0), in a) Kampong Speu, b) Kratie and c) Takeo provinces, Cambodia, 2004–2015. b = number of previous years to include; w = for the previous year, the number of weeks to include around the week we are predicting; w0 = for current year the number of previous week to include. A red triangle appears when the number of cases crosses the upper limit of the predictive curve. (TIF) [file pone.0212003.s003.TIF]
